# Association between low-density lipoprotein cholesterol and coronary plaque progression in non-dialysis chronic kidney disease: a retrospective cohort study

**DOI:** 10.1080/0886022X.2025.2594265

**Published:** 2025-12-10

**Authors:** Mengmeng Shan, Jie Li, Shaomi Li, Daopeng Dai, Suling Ye, Jiwei Yu, Shiqun Sun, Zhengbin Zhu, Ruiyan Zhang, Jinzhou Zhu

**Affiliations:** ^a^ Department of Internal Medicine, Ruijin-Hainan Hospital, Shanghai Jiao Tong University School of Medicine (Hainan Boao Research Hospital), Qionghai, China; ^b^ Department of Vascular & Cardiology, Ruijin Hospital, Shanghai Jiao Tong University School of Medicine, Shanghai, China

**Keywords:** Coronary plaque progression, chronic kidney disease, low-density lipoprotein cholesterol, estimated glomerular filtration rate

## Abstract

This study aimed to investigate the characteristics of coronary plaque progression in non-dialysis chronic kidney disease (CKD) patients, particularly focusing on the association between low-density lipoprotein cholesterol (LDL-C) levels and plaque progression across different stages of CKD. Two hundred ninety-two patients with CKD who underwent percutaneous coronary intervention during their initial coronary angiography and received follow-up angiography within 2 years were enrolled. These patients were classified into plaque progression and non-progression groups using quantitative coronary angiography analysis. Our findings demonstrate that patients with coronary plaque progression exhibited higher LDL-C levels and lower estimated glomerular filtration rate (eGFR). Smooth curve and logistic regression analysis revealed a gradual increase in the probability of plaque progression with elevated LDL-C levels, each 1 mmol/L increase in LDL-C was associated with a 2.63-fold higher risk of plaque progression in patients with eGFR of 45–60 mL/min/1.73 m^2^ (OR = 3.63, 95% CI: 1.65–8.01), and 2.44-fold increased risk in patients with eGFR <45 mL/min/1.73 m^2^ (OR = 3.44, 95% CI: 1.23–9.61). Kaplan–Meier curve and multivariate regression analysis demonstrated that maintaining LDL-C levels below the guideline-recommended very-high-risk threshold (1.4 mmol/L) conferred persistent protective effects against coronary plaque progression in CKD patients. This study demonstrated a graded association between LDL-C levels and coronary plaque progression in non-dialysis CKD patients, and this association was significantly weaker in the subgroup with lower eGFR compared to that with higher eGFR. Moreover, our findings suggest an association between lower LDL-C levels and reduced risk of coronary plaque progression in this high-risk population.

## 1. Introduction

Chronic kidney disease (CKD), as defined by the updated Kidney Disease: Improving Global Outcomes (KDIGO) guideline, is characterized by persistent structural or functional kidney abnormalities, evidenced by markers of kidney damage or glomerular filtration rate (GFR) <60 mL/min/1.73 m^2^ for ≥3 months [1]. CKD is strongly associated with accelerated cardiovascular disease (CVD), the leading cause of morbidity and mortality in this population [2,3]. Cardiovascular manifestations in CKD patients encompass heart failure, myocardial fibrosis, arterial stiffness, vascular calcification, and atherosclerosis [4,5]. Notably, atherosclerosis and atherosclerotic CVD represent critical pathophysiological features of CKD-related cardiovascular morbidity [6]. Epidemiological and histopathological studies since 1974 have demonstrated a higher prevalence of advanced atherosclerotic lesions in CKD patients compared to non-CKD controls [7,8], with the incidence and severity of coronary artery disease inversely correlating with declining GFR [9]. Experimental models, including ApoE-deficient mice, further corroborate CKD’s role in exacerbating atherosclerosis [10].

The pathogenesis of CKD-associated atherosclerosis involves both classical cardiovascular risk factors (e.g., diabetes, hypertension, dyslipidemia) and CKD-specific non-classical drivers. However, traditional risk stratification tools based on classical factors inadequately predict cardiovascular outcomes in CKD populations [11,12], suggesting a distinct pathophysiology. Emerging evidence implicates chronic inflammation, oxidative stress, endothelial dysfunction, calcium-phosphate dysregulation, uremic toxins, and hyperactivation of the renin–angiotensin system as key contributors to atherosclerosis in CKD [13,14]. Despite progress, the mechanistic interplay between CKD and atherogenesis remains incompletely understood.

Dyslipidemia, a well-established traditional risk factor for atherosclerosis, is highly prevalent among patients with CKD. The hallmark lipoprotein profile in CKD includes elevated levels of triglyceride (TG), very low-density lipoprotein cholesterol (VLDL-C), and remnant lipoproteins, reduced levels of high-density lipoprotein cholesterol (HDL-C), and less frequently increased low-density lipoprotein cholesterol (LDL-C) and total cholesterol (TC) levels [15]. This dyslipidemia arises through multiple CKD-specific mechanisms. Impaired lipoprotein lipase activity secondary to elevated apolipoprotein C-III levels disrupts chylomicron clearance, leading to TG accumulation [16,17]. The change of decreased HDL in CKD is primarily attributable to hypoalbuminemia and decreased expression of essential HDL constituents including apolipoprotein A. CKD induces hepatic HMG-CoA reductase upregulation and LDL receptor dysfunction through lysosomal impairment, creating a ‘double-hit’ mechanism that elevates LDL-C and TC [18,19]. Nephrotic-range proteinuria promotes hepatic Lp(a) overproduction while reducing renal clearance, creating a pro-atherogenic milieu [20]. Beyond quantitative lipid abnormalities, CKD induces qualitative lipoprotein modifications through oxidative, glycation, and carbamylation processes. These structural alterations: (1) enhance recognition by scavenger receptors; (2) convert HDL from anti-inflammatory to pro-oxidant states; and (3) generate advanced glycation end-products that activate pro-atherogenic signaling pathways [21–23]. Our previous study also revealed that tyrosine sulfation is enhanced in CKD and this may be associated with CKD-related atherosclerosis [24]. This multilayered dysregulation-encompassing lipid metabolism derangements, pathogenic lipoprotein modifications, and receptor signaling alterations creates a self-perpetuating cycle of vascular injury in CKD.

In addition to increased atherosclerosis burden, clinical studies highlight accelerated coronary plaque progression in CKD patients compared to those with preserved renal function [25–27]. However, the pathophysiological drivers of plaque dynamics across CKD stages remain poorly characterized. This retrospective cohort study enrolled CKD patients who received follow-up coronary angiography after percutaneous coronary intervention (PCI). Using quantitative coronary angiography (QCA), we assessed coronary plaque progression and investigated CKD-specific risk factors associated with plaque progression. Addressing current controversies surrounding lipid management in CKD [28–30], we systematically investigate the nonlinear dose-response relationship between LDL-C trajectories and plaque progression kinetics, incorporating stratified analyses by GFR. By establishing CKD-stage LDL-C thresholds predictive of rapid plaque expansion, our research aims to establish an evidence base for developing lipid-lowering strategies specifically tailored to this distinct patient population with CKD.

## 2. Methods

### 2.1. Patients and ethical statement

**Figure 1.**
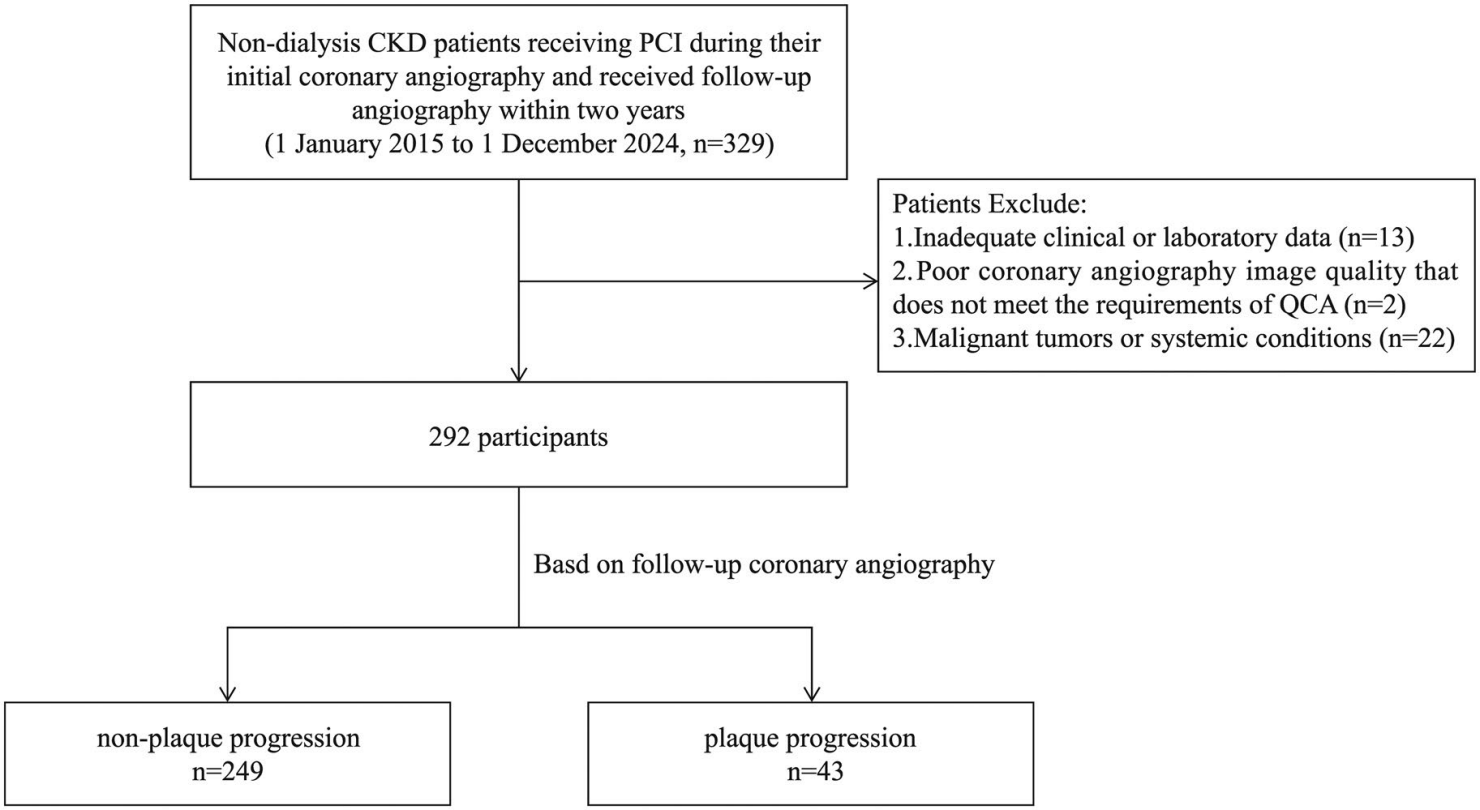
Flow chart of patient enrollment. CKD, chronic kidney disease; PCI, percutaneous coronary intervention; QCA, quantitative coronary angiography.

This retrospective cohort study enrolled 292 non-dialysis CKD patients [estimated glomerular filtration rate (eGFR) < 60 mL/min/1.73 m^2^ persisting for ≥3 months] hospitalized at Ruijin Hospital in Shanghai, China, from January 2015 to December 2024, with eGFR calculated through the Chronic Kidney Disease Epidemiology Collaboration (CKD-EPI) equation [31]. All enrolled participants underwent PCI during their initial angiography and received follow-up angiography within 2 years. Exclusion criteria involved missing data for analysis, poor angiography image quality that does not meet the requirements of QCA, malignant tumors or systemic conditions (Figure 1). The study protocol received approval from the Ethics Committee of Ruijin Hospital, Shanghai (Ethics ID: 2015-109). All procedures were performed in accordance with the Declaration of Helsinki, and written informed consent was obtained from all participants.

### 2.2. Assessment of coronary artery plaque progression

Coronary angiography procedures were performed via the radial artery using the Judkins method, with each coronary artery visualized in at least two orthogonal angiographic projections. All angiograms underwent standardized semi-automated QCA analysis (GE Healthcare, UK) with catheter calibration to measure minimal lumen diameter (MLD). Subsequently, two interventional cardiologists independently performed blinded assessments of the DICOM-archived images. In the case of disagreement, the opinion of a senior interventional cardiologist was sought, and the final decision was made by consensus. This tripartite approach integrating automated quantification, double-blind review, and concordant arbitration thereby minimized inter-observer variability. Follow-up angiography was subsequently conducted to evaluate changes in MLD, with the percentage change in MLD calculated accordingly.

Plaque progression was defined according to previous studies [32,33] and our prior research publication [34]: (1) ≥10% reduction in the diameter of a preexisting lesion with >50% stenosis; (2) increase of ≥30% reduction in the diameter of a preexisting lesion with <50% stenosis; (3) the development of a new stenosis with *a* ≥ 30% reduction in the diameter of a segment that was normal on the first angiogram, or the progression of any lesion to total occlusion on follow-up coronary angiography.

### 2.3. Data collection

Demographic, clinical, and laboratory data for all participants were obtained from the Ruijin Hospital’s Hospital Information System, encompassing baseline characteristics [age, sex, body mass index (BMI), coronary angiography follow-up interval], cardiovascular risk factors (hypertension, current smoking, current alcohol use), revascularization history [prior myocardial infarction (MI), prior PCI, prior coronary artery bypass grafting (CABG)], and diagnostic outputs from biochemical assays, urinary biomarkers, and echocardiography. Following a 12-h fasting period, morning venous blood and midstream urine specimens were collected for centralized analysis. Biochemical analyses were performed using standardized protocols on Hitachi 7600 and Roche Modular platforms (Hitachi Ltd, Tokyo, Japan), covering: (1) hematologic indices: white blood cells (WBCs), neutrophil count, hemoglobin (HGB), platelets (PLTs); (2) inflammation: c-reactive protein (CRP); (3) organ function: alanine aminotransferase (ALT), aspartate aminotransferase (AST), albumin (ALB), creatinine, blood urea nitrogen (BUN), cystatin C, uric acid (UA); (4) metabolic metrics: glycated hemoglobin (HbA1C), LDL-C, HDL-C, TG; (5) cardiac biomarkers: creatine kinase-myocardial band (CK-MB), cardiac troponin I (cTnI), B-type natriuretic peptide (BNP); (6) coagulation profile: activated partial thromboplastin time (APTT), prothrombin time (PT), D-dimer; (7) electrolyte: calcium, phosphorus. Urinary assessment included albumin–creatinine ratio (ACR). Left ventricular ejection fraction (LVEF) was measured and recorded during echocardiography. All variables were captured under a standardized protocol during the second scheduled angiographic follow-up.

### 2.4. Statistical analysis

Categorical variables were expressed as number of cases (percentage), continuous variables as mean ± standard deviation (SD). Differences in categorical variables between the coronary plaque progression and non-progression groups were compared using the chi-square test, continuous variables were analyzed using the Student’s *t*-test. Smoothed curves based on restricted cubic spline functions and logistic regression analysis were applied to evaluate the association trends between LDL-C and coronary plaque progression across varying GFR levels. Kaplan–Meier (KM) curves and regression analysis were used to assess whether maintaining LDL-C at lower levels may confer greater clinical benefits in non-dialysis CKD patients. Receiver operating characteristic (ROC) curves were plotted, and the area under the curve (AUC) was calculated. Prior to logistic regression, collinearity screening of covariates was performed, and all variance inflation factors were confirmed to be below 5. Data analysis was conducted using EmpowerXYS, with *P* < 0.05 considered statistically significant.

## 3. Results

### 3.1. Characteristics of the study population

This study included 292 non-dialysis CKD patients, among whom 43 developed coronary plaque progression. Compared to those without plaque progression, patients with plaque progression exhibited higher levels of WBCs, PLTs, HbA1C, creatinine, BUN, ACR, and lower levels of calcium. Notably, CKD patients with plaque progression had elevated LDL-C levels and reduced eGFR levels. Detailed baseline characteristics are summarized in Table 1.

**Table 1.** Characteristics of the study population.

	Non-plaque progression (*n* = 249)	Plaque progression (*n* = 43)
Follow-up interval (months)	14.98 ± 4.46	17.47 ± 5.12
Age (years)	74.28 ± 7.58	71.86 ± 10.25
Sex (male%)	170 (68.27%)	32 (74.42%)
Current smoking (%)	20 (8.03%)	5 (11.63%)
Current alcohol use (%)	8 (3.21)	1 (2.33)
Hypertension (%)	203 (81.53%)	35 (81.40%)
Previous MI	0 (0.00)	1 (2.33)
Previous PCI	47 (18.88)	4 (9.30)
Previous CABG	6 (2.41)	1 (2.33)
Statin	245 (98.39)	42 (97.67)
Atorvastatin	183 (73.49)	30 (69.77)
Rosuvastatin	54 (21.69)	12 (27.91)
Pravastatin	6 (2.41)	0 (0.00)
Pivastatin	1 (0.40)	0 (0.00)
Simvastatin	1 (0.40)	0 (0.00)
BMI (kg/m^2^)	24.80 ± 3.24	25.14 ± 3.74
WBCs (10^9^/L)	6.11 ± 1.69	6.70 ± 1.74
Neutrophil (%)	61.44 ± 8.88	63.07 ± 7.12
HGB (g/L)	124.28 ± 16.82	123.49 ± 20.21
PLTs (10^9^/L)	175.65 ± 50.99	199.81 ± 55.32
CRP (mg/L)	3.56 ± 11.24	5.93 ± 10.61
ALT (IU/L)	20.97 ± 23.49	17.81 ± 7.20
AST (IU/L)	23.12 ± 13.51	20.42 ± 5.14
ALB (g/L)	37.73 ± 3.41	36.95 ± 3.24
APTT (s)	29.59 ± 3.08	29.71 ± 2.80
PT (s)	11.71 ± 1.18	11.75 ± 1.85
D-dimer (mg/L)	0.62 ± 0.88	0.61 ± 0.61
Calcium (mmol/L)	2.24 ± 0.12	2.19 ± 0.15
Phosphorus (mmol/L)	1.14 ± 0.21	1.17 ± 0.26
HbA1C (%)	6.67 ± 1.12	7.12 ± 1.83
CK-MB (ng/mL)	2.47 ± 5.16	2.37 ± 1.54
cTnI (pg/mL)	63.92 ± 358.84	178.29 ± 613.63
BNP (pg/mL)	1,082.13 ± 3,572.50	1,526.05 ± 5,404.51
LVEF (%)	63.35 ± 8.16	61.28 ± 9.66
UA (μmol/L)	405.46 ± 96.40	411.70 ± 102.40
Creatinine (μmol/L)	122.00 ± 43.30	155.67 ± 110.01
BUN (mmol/L)	8.53 ± 2.89	9.60 ± 4.24
CysC (mg/L)	1.79 ± 2.32	1.90 ± 0.72
ACR (mg/mmoL)	19.17 ± 60.76	47.44 ± 90.64
eGFR (mL/min/1.73 m^2^)	48.64 ± 9.38	42.99 ± 13.20
TG (mmol/L)	1.58 ± 1.32	1.74 ± 1.09
HDL-C (mmol/L)	1.10 ± 0.28	1.10 ± 0.45
LDL-C (mmol/L)	1.75 ± 0.61	2.29 ± 0.78
LDL-C level		
>1.8 mmol/L	93 (37.35)	25 (58.14)
1.4–1.8 mmol/L	84 (33.73)	16 (37.21)
<1.4 mmol/L	72 (28.92)	2 (4.65)

*Notes:* Results are expressed as mean ± SD, median (quartile), or as number (frequency) for binary variables.

*Abbreviations:* MI: myocardial infarction; PCI: percutaneous coronary intervention; CABG: coronary artery bypass grafting; BMI: body mass index; WBCs: white blood cells; HGB: hemoglobin; PLTs: platelets; CRP: c-reactive protein; ALT: alanine aminotransferase; AST: aspartate aminotransferase; ALB: albumin; APTT: activated partial thromboplastin time; PT: prothrombin time; HbA1C: glycated hemoglobin; CK-MB: creatine kinase-myocardial band; cTnI: cardiac troponin I; BNP: B-type natriuretic peptide; LVEF: left ventricular ejection fractions; UA: uric acid; BUN: blood urea nitrogen; CysC: cystatin C; ACR: albumin–creatinine ratio; eGFR: estimated glomerular filtration rate; TG: triglyceride; HDL-C: high-density lipoprotein cholesterol; LDL-C: low-density lipoprotein cholesterol.

### 3.2. Association between LDL-C and coronary plaque progression

**Figure 2.**
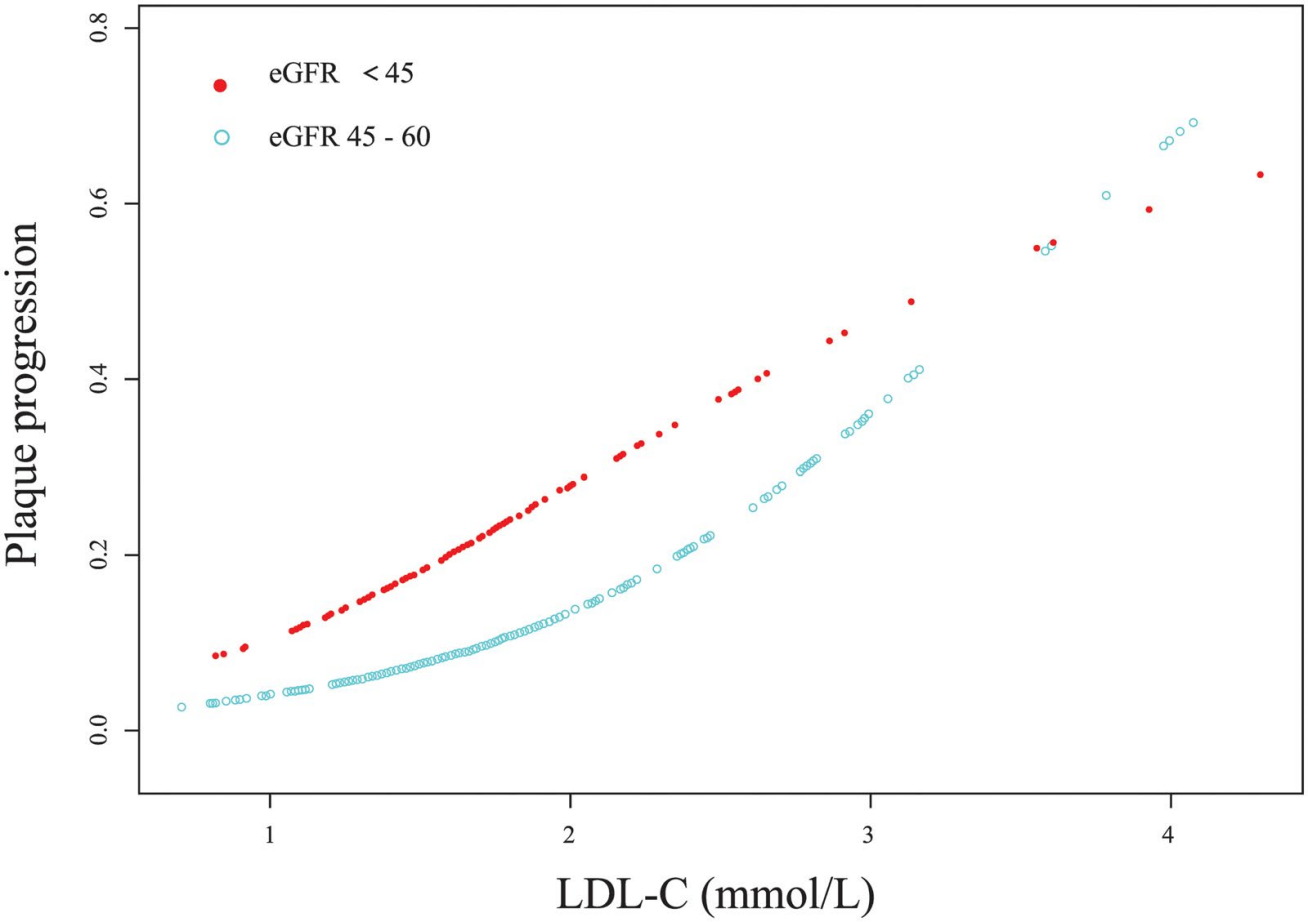
LDL-C smooth curves stratified by eGFR (<45 vs. 45–60 mL/min/1.73 m^2^) in relation to coronary plaque progression.

Smooth curve analysis revealed a gradual increase in the risk of coronary plaque progression with elevated LDL-C levels, though this relationship was not as significant in eGFR <45 mL/min/1.73 m^2^ as in eGFR 45–60 mL/min/1.73 m^2^ (Figure 2). Logistic regression analysis demonstrated that for participants with eGFR 45–60 mL/min/1.73 m^2^, every 1 mmol/L increase in LDL-C was associated with a 2.63-fold higher risk of plaque progression (OR = 3.63, 95% CI: 1.65–8.01, *P* = 0.0014), while among patients with eGFR <45 mL/min/1.73 m^2^, the same LDL elevation corresponded to a 2.44-fold increased risk of plaque progression (OR = 3.44, 95% CI: 1.23–9.61, *P* = 0.0181). Detailed results of the logistic regression analyses are presented in Table 2.

**Table 2.** Logistic regression analysis of LDL-C and coronary plaque progression.

	OR (95% CI) *P*					
	eGFR < 45 mL/min/1.73 m^2^		eGFR 45–60 mL/min/1.73 m^2^		Total (eGFR 0–60 mL/min/1.73 m^2^)	
	Single factor	Multivariate	Single factor	Multivariate	Single factor	Multivariate
Follow-up interval	1.11 (1.00, 1.23) 0.0399	1.08 (0.96, 1.22) 0.2071	1.12 (1.02, 1.22) 0.0140	1.06 (0.95, 1.19) 0.2860	1.11 (1.04, 1.19) 0.0015	1.08 (1.00, 1.16) 0.0550
Sex	1.35 (0.46, 3.94) 0.5847		1.53 (0.54, 4.33) 0.4262		1.35 (0.65, 2.82) 0.4215	
Age	0.96 (0.91, 1.02) 0.1741		0.97 (0.91, 1.02) 0.1941		0.96 (0.93, 1.00) 0.0712	
Current smoking	3.83 (0.50, 29.11) 0.1939		1.33 (0.36, 4.93) 0.6663		1.51 (0.53, 4.26) 0.4392	
Current alcohol use	1.19 (0.12, 12.14) 0.8815		inf. (0.00, inf.) inf.		0.72 (0.09, 5.88) 0.7569	
Hypertension	0.55 (0.17, 1.82) 0.3284		1.63 (0.46, 5.80) 0.4494		0.99 (0.43, 2.28) 0.9837	
Previous MI	inf. (0.00, inf.) inf.		inf. (0.00, inf.) inf.		inf. (0.00, inf.) inf.	
Previous PCI	0.50 (0.10, 2.41) 0.3840		0.40 (0.09, 1.80) 0.2348		0.44 (0.15, 1.29) 0.1360	
Previous CABG	inf. (0.00, inf.) inf.		1.57 (0.18, 14.09) 0.6856		0.96 (0.11, 8.21) 0.9735	
Statin	0.55 (0.05, 6.41) 0.6337		inf. (0.00, inf.) inf.		0.69 (0.07, 6.29) 0.7386	
BMI	1.10 (0.96, 1.26) 0.1808		0.97 (0.84, 1.11) 0.6158		1.03 (0.94, 1.14) 0.5341	
WBCs	1.13 (0.89, 1.44) 0.3062	1.26 (0.85, 1.88) 0.2562	1.21 (0.93, 1.57) 0.1650	0.78 (0.52, 1.16) 0.2214	1.20 (1.01, 1.43) 0.0369	0.99 (0.76, 1.28) 0.9150
Neutrophil	0.99 (0.94, 1.05) 0.7492		1.04 (0.98, 1.09) 0.1743		1.02 (0.98, 1.06) 0.2541	
HGB	1.00 (0.97, 1.03) 0.9940		1.01 (0.98, 1.05) 0.3710		1.00 (0.98, 1.02) 0.7824	
PLTs	1.01 (1.00, 1.01) 0.1510	1.01 (0.99, 1.02) 0.2955	1.01 (1.00, 1.02) 0.0188	1.02 (1.00, 1.03) 0.0047	1.01 (1.00, 1.01) 0.0071	1.01 (1.00, 1.02) 0.0185
CRP	1.01 (0.97, 1.05) 0.6703		1.01 (0.99, 1.04) 0.3112		1.01 (0.99, 1.04) 0.2408	
ALT	0.93 (0.85, 1.01) 0.0661		1.01 (0.97, 1.05) 0.5166		0.98 (0.95, 1.02) 0.3309	
AST	0.94 (0.86, 1.02) 0.1300		0.99 (0.92, 1.05) 0.6706		0.96 (0.91, 1.01) 0.1584	
TP	0.96 (0.88, 1.05) 0.3665		1.03 (0.94, 1.12) 0.5718		0.99 (0.93, 1.06) 0.8446	
ALB	0.91 (0.79, 1.04) 0.1697		1.00 (0.87, 1.14) 0.9516		0.93 (0.85, 1.03) 0.1659	
APTT	0.99 (0.85, 1.16) 0.9337		1.06 (0.92, 1.22) 0.4455		1.01 (0.91, 1.12) 0.8191	
PT	1.02 (0.59, 1.76) 0.9509		1.04 (0.79, 1.36) 0.7910		1.03 (0.81, 1.30) 0.8277	
D-dimer	0.71 (0.26, 1.94) 0.5057		1.20 (0.68, 2.13) 0.5297		0.98 (0.66, 1.46) 0.9297	
Calcium	0.06 (0.00, 1.60) 0.0933	0.03 (0.00, 6.60) 0.2019	0.10 (0.00, 4.96) 0.2478	0.03 (0.00, 5.17) 0.1766	0.06 (0.01, 0.78) 0.0313	0.04 (0.00, 1.26) 0.0677
Phosphorus	1.37 (0.27, 6.92) 0.7012		0.83 (0.06, 10.90) 0.8866		1.72 (0.45, 6.61) 0.4284	
HbA1C	1.07 (0.79, 1.46) 0.6563	0.93 (0.61, 1.42) 0.7440	1.51 (1.07, 2.14) 0.0189	1.65 (1.03, 2.64) 0.0366	1.28 (1.02, 1.61) 0.0320	1.21 (0.91, 1.60) 0.1952
CK-MB	0.96 (0.84, 1.10) 0.5889		1.17 (0.90, 1.52) 0.2507		1.00 (0.92, 1.07) 0.9043	
cTnI	1.00 (1.00, 1.00) 0.5807	1.00 (1.00, 1.00) 0.1767	1.00 (1.00, 1.00) 0.0170	1.00 (1.00, 1.01) 0.2388	1.00 (1.00, 1.00) 0.1253	1.00 (1.00, 1.00) 0.9069
BNP	1.00 (1.00, 1.00) 0.9803		1.00 (1.00, 1.00) 0.4903		1.00 (1.00, 1.00) 0.4964	
LVEF	1.03 (0.96, 1.10) 0.3624	1.07 (0.98, 1.18) 0.1379	0.95 (0.91, 0.99) 0.0192	0.94 (0.89, 0.99) 0.0306	0.97 (0.94, 1.01) 0.1406	0.98 (0.94, 1.02) 0.3626
UA	1.00 (1.00, 1.01) 0.9179		1.00 (1.00, 1.00) 0.9497		1.00 (1.00, 1.00) 0.6971	
Creatinine	1.01 (1.00, 1.01) 0.0794	1.01 (0.98, 1.03) 0.6523	1.01 (0.98, 1.04) 0.3756	1.03 (0.98, 1.08) 0.2081	1.01 (1.00, 1.01) 0.0161	1.01 (0.99, 1.02) 0.3444
BUN	1.07 (0.96, 1.20) 0.2367	0.94 (0.70, 1.24) 0.6486	0.96 (0.75, 1.22) 0.7242	0.86 (0.61, 1.21) 0.3928	1.09 (1.00, 1.19) 0.0477	0.86 (0.70, 1.04) 0.1258
Cystatin C	0.98 (0.83, 1.16) 0.8168		1.38 (0.25, 7.75) 0.7157		1.02 (0.90, 1.15) 0.7628	
ACR	1.00 (1.00, 1.01) 0.2285	1.00 (0.99, 1.00) 0.2486	1.01 (1.00, 1.02) 0.1229	1.00 (0.98, 1.01) 0.7942	1.00 (1.00, 1.01) 0.0187	1.00 (0.99, 1.00) 0.3022
eGFR	0.93 (0.88, 0.99) 0.0190	0.93 (0.78, 1.10) 0.3756	0.98 (0.89, 1.08) 0.6931	0.98 (0.85, 1.13) 0.7629	0.95 (0.93, 0.98) 0.0013	0.94 (0.89, 1.00) 0.0354
TG	1.02 (0.79, 1.32) 0.8674		1.11 (0.71, 1.73) 0.6392		1.09 (0.88, 1.35) 0.4479	
HDL-C	0.42 (0.05, 3.74) 0.4344		1.54 (0.50, 4.74) 0.4552		1.00 (0.35, 2.86) 0.9995	
LDL-C	2.40 (1.15, 4.98) 0.0194	3.44 (1.23, 9.61) 0.0181	3.60 (1.97, 6.60) <0.0001	3.63 (1.65, 8.01) 0.0014	2.86 (1.82, 4.48) <0.0001	3.05 (1.76, 5.28) <0.0001

*Abbreviations:* LDL-C: low-density lipoprotein cholesterol; eGFR: estimated glomerular filtration rate; MI: myocardial infarction; PCI: percutaneous coronary intervention; CABG: coronary artery bypass grafting; BMI: body mass index; WBCs: white blood cells; HGB: hemoglobin; PLTs: platelets; CRP: c-reactive protein; ALT: alanine aminotransferase; AST: aspartate aminotransferase; TP: total protein; ALB: albumin; APTT: activated partial thromboplastin time; PT: prothrombin time; HbA1C: glycated hemoglobin; CK-MB: creatine kinase-myocardial band; cTnI: cardiac troponin I; BNP: B-type natriuretic peptide; LVEF: left ventricular ejection fractions; UA: uric acid; BUN: blood urea nitrogen; ACR: albumin–creatinine ratio; TG: triglyceride; HDL-C: high-density lipoprotein cholesterol.

**Figure 3.**
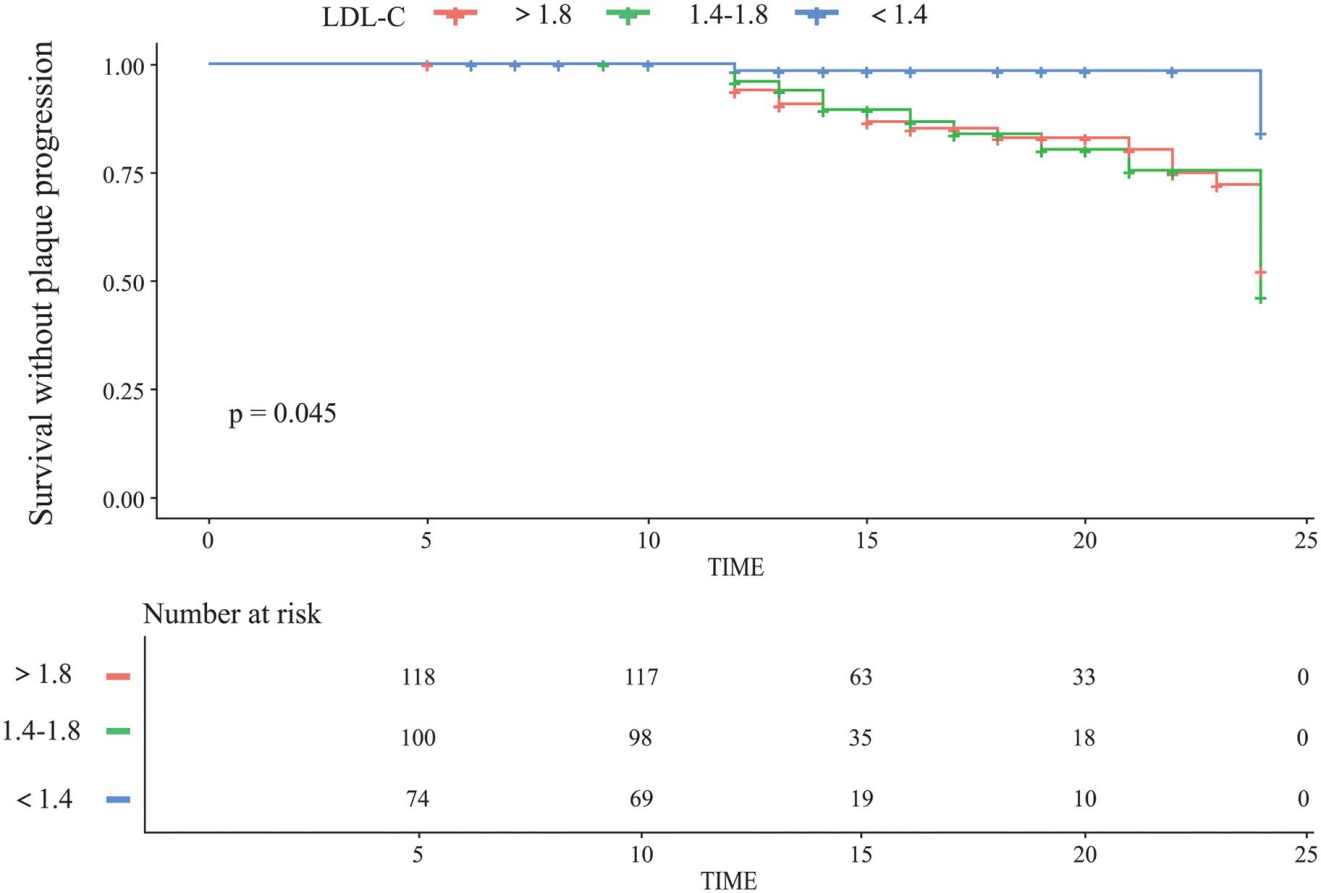
KM curve of LDL-C level and survival probability without plaque progression.

Current evidence-based guidelines recommend maintaining LDL-C levels below 1.8 mmol/L for patients with established coronary heart disease, while suggesting a more stringent target of below 1.4 mmol/L for those at very high cardiovascular risk [28,29]. Given the lack of specific thresholds for cardiovascular-kidney disease comorbidity, we applied these targets to our cohort. The analysis, which adjusted for 4 independent risk factors in multivariate regression (Adjusted 1) and 11 risk factors in univariate regression (Adjusted 2), revealed that maintaining LDL-C levels below 1.4 mmol/L was associated with a significant reduction in coronary plaque progression risk, independent of eGFR status (Table 3). KM survival analysis further corroborated these findings: Participants with LDL-C controlled below 1.4 mmol/L exhibited improved event-free survival rates compared to those with LDL-C levels of 1.4–1.8 or >1.8 mmol/L (Figure 3).

**Table 3.** Logistic regression analysis of LDL-C level and coronary plaque progression in different stage of CKD.

		Non-adjusted	Adjusted 1	Adjusted 2
		OR (95%CI) *P*	OR (95% CI) *P*	OR (95% CI) *P*
eGFR < 45	LDL-C > .8	Ref.	Ref.	Ref.
	LDL-C 1.4–1.8	0.79 (0.27, 2.30) 0.6685	0.82 (0.25, 2.71) 0.7467	0.58 (0.15, 2.20) 0.4214
	LDL-C < 1.4	0.09 (0.01, 0.78) 0.0283	0.07 (0.01, 0.99) 0.0487	0.04 (0.01, 0.71) 0.0280
eGFR 45–60	LDL-C > 1.8	Ref.	Ref.	Ref.
	LDL-C 1.4–1.8	0.56 (0.21, 1.47) 0.2394	0.61 (0.22, 1.69) 0.3419	0.67 (0.21, 2.12) 0.4914
	LDL-C < 1.4	0.10 (0.01, 0.77) 0.0273	0.09 (0.01, 0.75) 0.0258	0.11 (0.01, 0.97) 0.0474
eGFR 0–60	LDL-C > 1.8	Ref.	Ref.	Ref.
	LDL-C 1.4–1.8	0.71 (0.35, 1.42) 0.3302	0.74 (0.35, 1.55) 0.4191	0.81 (0.37, 1.78) 0.6057
	LDL-C < 1.4	0.10 (0.02, 0.45) 0.0025	0.09 (0.02, 0.43) 0.0023	0.11 (0.02, 0.53) 0.0059

*Abbreviations:* eGFR (mL/min/1.73 m^2^): estimated glomerular filtration rate; LDL-C (mmol/L): low-density lipoprotein cholesterol; CKD: chronic kidney disease.

Non-adjusted: no confounding factors were adjusted. Adjusted 1: adjusted for PLTs, HbA1C, LVEF, and eGFR. Adjusted 2: adjusted for follow-up interval, WBCs, PLTs, calcium, HbA1C, cTnI, LVEF, creatinine, BUN, ACR, and eGFR.

### 3.3. The ability of LDL-C to evaluate the progression of coronary plaque

**Figure 4.**
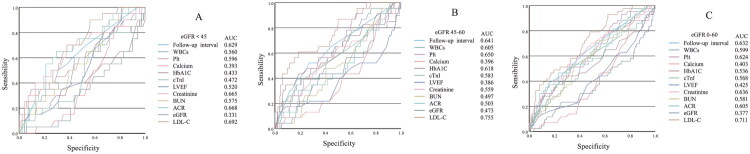
ROC curve in different eGFR level.

Multivariate ROC analysis integrating all coronary plaque progression-related variables identified serum LDL-C as the strongest independent predictor of atherosclerotic plaque dynamics across all renal function strata defined by eGFR levels (eGFR <45 mL/min/1.73 m^2^, AUC = 0.692; eGFR 45–60 mL/min/1.73 m^2^, AUC = 0.755; eGFR 0–60 mL/min/1.73 m^2^, AUC = 0.711) (Figure 4).

## 4. Discussion

The well-documented acceleration of atherosclerosis in CKD populations has established CKD as an independent cardiovascular risk factor in major guidelines [28,29]. Crucially, the graded increase in atherosclerosis risk across CKD stages underscores the intrinsic atherogenicity of progressive renal dysfunction [35]. Current evidence remains sparse regarding atherosclerotic risk stratification in CKD populations, while more critically, there persists clinical equipoise surrounding evidence-based LDL-C target thresholds for atherosclerosis prevention [30].

In the present retrospective cohort study, multivariable logistic regression identified eGFR and LDL-C as independent predictors of rapid plaque progression in non-dialysis CKD patients. Notably, we observed a graded association between LDL-C levels and coronary plaque progression in non-dialysis CKD patients, and this association was significantly weaker in the subgroup with lower eGFR compared to that with higher eGFR. The AUC values (0.692–0.755) indicate moderate rather than strong discriminatory power, but given the complex pathophysiology of CKD, a single biomarker is unlikely to fully capture plaque progression risk. Nonetheless, LDL-C demonstrated comparatively stronger predictive ability relative to other clinical factors examined. Moreover, our findings suggest that intensive LDL-C reduction may play an important clinical role in preventing coronary plaque progression among CKD patients. Cardiovascular protection persists in CKD patients even under the guideline-recommended LDL-C threshold of 1.4 mmol/L for very-high-risk stratification.

Coronary plaque progression refers to a quantifiable increase in plaque burden within the coronary arteries. Intracoronary imaging modalities such as intravascular ultrasound (IVUS), optical coherence tomography (OCT), and near-infrared spectroscopy (NIRS) enable more detailed observation of plaque characteristics and subtle changes, thereby providing a more accurate assessment of progression. However, their utilization is limited by factors including healthcare costs and procedural invasiveness. In this study, we defined plaque progression through longitudinal changes in MLD measured by QCA at baseline and follow-up, consistent with our prior methodology [34]. Coronary computed tomography angiography (CCTA) is another important noninvasive alternative to coronary angiography for evaluating coronary plaque progression [36,37]. However, severe calcification and artifacts may compromise the accuracy of stenosis assessment. Crucially, the inability to personalize contrast dosing in standard CTA protocols elevates contrast-induced nephropathy risk in this vulnerable cohort [38,39].

Dyslipidemia in CKD patients manifests as a multifaceted metabolic derangement characterized by both quantitative and qualitative lipid abnormalities. This unique pathological profile suggests that lipid-lowering strategies for this population may require distinct approaches compared to those validated in the general population. While statins remain first-line agents for LDL-C reduction in non-dialysis CKD, demonstrating 28% relative risk reduction in major CVD events [40,41], their benefits paradoxically dissipate in dialysis-dependent patients, as evidenced by the SHARP trial where simvastatin/ezetimibe combination reduced non-fatal MI, ischemic stroke, and coronary revascularization in mild-to-moderate CKD but showed neutral effects in dialysis subgroups [42]. Emerging PCSK9 inhibitors exhibit consistent LDL-C lowering across CKD stages in FOURIER/ODYSSEY trials [43,44], yet their exclusion of severe CKD and dialysis patients precludes definitive safety/efficacy conclusions in advanced CKD. Current guidelines reflect this uncertainty: AHA/ACC 2019 and KDIGO 2024 advocates risk-based statin initiation in non-dialysis CKD without fixed LDL-C targets [1,29], whereas ESC/EAS 2019 stratifies severe CKD (eGFR < 30 mL/min/1.73 m^2^) as very high risk, mandating LDL-C <1.4 mmol/L [28]. Our longitudinal analysis validates the European LDL-C threshold by demonstrating a 96% reduction in plaque progression risk (OR = 0.04, 95% CI: 0.01–0.71, *P* = 0.0280) among patients with eGFR <45 mL/min/1.73 m^2^ achieving LDL-*C* < 1.4 mmol/L, with progressively higher hazards observed at LDL-C levels of 1.4–1.8 and >1.8 mmol/L. These findings underscore the necessity for CKD-stage-stratified randomized controlled trials to address current guideline inconsistencies and advance personalized lipid management strategies that account for both quantitative lipoprotein targets and CKD-specific qualitative modifications.

CKD constitutes a distinct clinical syndrome wherein atherosclerosis is driven by both traditional risk factors and CKD-specific pathological amplifiers. Beyond traditional cardiovascular risks, the CKD continuum fosters a unique pro-atherogenic triad: (1) sustained systemic inflammation evidenced by elevated CRP and IL-6 [45]; (2) oxidative stress amplification via nicotinamide adenine dinucleotide phosphate oxidase-2 upregulation [46]; (3) immune dysregulation characterized by monocyte expansion and endotoxin-tolerant macrophage phenotypes [47]. Crucially, these nontraditional drivers exhibit stage-dependent escalation—severe CKD demonstrates 4.2-fold higher IL-6 and 3.6-fold greater F2-isoprostanes than moderate CKD [45], mechanistically explaining the attenuated efficacy of isolated LDL-C lowering in advanced stages [42]. This pathophysiological complexity undermines conventional risk prediction, rendering Framingham scores insensitive in CKD due to unaccounted inflammatory-uremic variables. Our investigation quantitatively aligns with these findings, which indicated that the association between LDL-C and plaque progression was weaker among patients with lower eGFR compared to those with higher eGFR. That does not mean that lipid-lowering treatment have less beneficial effect CKD patients as GFR declined, but this benefit might be masked by stronger inflammatory response. In this situation, aggressive LDL-C reduction may constitute an effective plaque-stabilization strategy, as quantitatively validated in our study, which ‌showed that maintaining LDL-C levels below 1.4 mmol/L significantly prevent coronary plaque progression in patients with CKD. Yet, less information is obtained if very low lipid levels could induce other complications. For instance, the most common treatment emergent adverse events seen in the PCSK9 inhibitor treated CKD patients from ODYSSEY clinical trials were nasopharyngitis, urinary tract infection, and upper respiratory infection. Considering the prevalent low immunity characteristic in CKD patients, it is possible that CKD patients would suffer severe infections after very low lipid treatment [44]. Thus, the effectiveness and safety of very low lipid level strategy in CKD patients should be studied in the future.

## 5. Limitations

This study has several limitations. Firstly, plaque progression was assessed using QCA, which evaluates luminal dimensions rather than direct plaque volume or composition. While QCA is widely used and clinically practical, it may not fully capture biological plaque progression compared to more advanced imaging techniques such as IVUS or OCT. Secondly, the study cohort was restricted to CKD patients who underwent PCI and completed follow-up angiography—a selected population that may not be representative of the broader CKD patients, which resulted in the exclusion of numerous CVD patients with concurrent renal impairment. This selection bias may increase the risk of type II statistical errors. Future large-scale, multi-center studies including more diverse demographic and clinical settings are warranted to validate and extend our results. Thirdly, as an observational study, no active intervention on LDL-C was implemented. Furthermore, LDL-C levels were measured at the time of follow-up angiography rather than at baseline, introducing ambiguity in the temporal sequence, thereby limiting our ability to establish causal relationships between lipid parameters and plaque progression dynamics. Finally, although we implemented covariate adjustment to mitigate potential confounders, residual confounding from unmeasured variables could persist and influence the interpretation of our findings.

## 6. Conclusion

In conclusion, our study delineates a CKD-specific atherogenic profile where eGFR and LDL-C variability exhibited predictive significance for coronary plaque progression. Importantly, LDL-C demonstrated divergent risk trajectories across CKD stages. We found that LDL-C levels associated with the progression of atherosclerosis disease differed as GFR declined, whereas the effect of LDL-C on coronary plaque progression decreased as GFR declined. More importantly, our study suggested strengthened low lipid therapy was needed in CKD patients in order to decrease the incidence of plaque progression. This study may help better understanding the complex underlying mechanism of hyperlipidemia under CKD condition.

## Data Availability

All data generated or analyzed during this study are openly available. Further enquiries can be directed to the corresponding author.
